# Association between hidradenitis suppurativa and obstructive sleep apnea: A retrospective cohort study using the TriNetX dataset

**DOI:** 10.1016/j.jdin.2025.04.010

**Published:** 2025-06-02

**Authors:** Mihir K. Patil, Thomas Z. Rohan, Goranit Sakunchotpanit, Kaushik P. Venkatesh, Sofia Milosavljevic, Vinod E. Nambudiri, Alexandra Charrow

**Affiliations:** a Department of Dermatology, Brigham and Women’s Hospital, Boston, Massachusetts; b Carle Illinois College of Medicine, University of Illinois Urbana-Champaign, Urbana, Illinois; c Sidney Kimmel Medical College, Philadelphia, Pennsylvania; d Tufts University School of Medicine; Boston, Massachusetts; e Harvard Medical School, Boston, Massachusetts

**Keywords:** clinical informatics, database, epidemiology, evidence-based medicine, general dermatology, hidradenitis suppurativa, immunopathology, inflammation/inflammatory, public health, quality of life, systemic diseases, TriNetX

*To the Editor:* Obstructive sleep apnea (OSA) – characterized by recurrent upper airway blockage from pharyngeal muscle hypotonia, transient hypoxic/apneic episodes causing nighttime awakening, daytime fatigue, and impaired cognition – affects about 20% of American adults.^1^ Prior research has demonstrated a potential association between OSA and hidradenitis suppurativa (HS),^2^^,^^3^ but routine screening for OSA is not currently recommended due to insufficient evidence.^4^ The pathophysiology of OSA is partially characterized by inflammation,^1^ which is significant in patients with HS.

To explore further, we utilized the TriNetX (Cambridge, MA) United States network and conducted a retrospective cohort study of patients ≥18 years with at least 2 HS diagnoses (*International Statistical Classification of Diseases and Related Health Problems*, *Tenth Revision* (ICD-10): L73.2, validated HS code)^2^ up to June 18^th^, 2024. Controls included adult patients with at least 2 general check-up encounters (Z00) at least 1 year apart and no HS history. OSA was identified via validated ICD-10 code G47.33.^2^ We performed unadjusted and adjusted analyses with 1:1 propensity score matching between cohorts for current age, sex, and race. Adjusted analysis utilized matching by OSA risk factors: obesity, smoking, hypertension, diabetes, and hyperlipidemia. Statistical analyses were performed in TriNetX with 95% confidence intervals and α = 0.05.

204,601 of 96,573,970 individuals met diagnostic criteria for HS. Of these, 97,472 had at least 2 diagnoses documented; 95,307 were included after matching to controls. Table I contains baseline cohort demographics. Individuals with HS were significantly more likely to have a diagnosis of OSA compared to those without HS; absolute OSA association was 13.5% (*n* = 12,889) and 9.3% (*n* = 8832) in the HS and control cohort, respectively (risk ratio: 2.02, 95% CI: 1.96-2.08, *P* < .0001) (Table II). After adjusting for OSA risk factors, the association remained elevated (risk ratio: 1.46, 95% CI 1.42-1.50, *P* < .0001).

**Table I:** Characteristics of patients with HS matched by age, sex, race, and risk factors for sleep apnea development

Characteristic	Before matching			After matching		
	Cases (*n* = 97,472)	Controls (*n* = 7,902,165)	*P*-value	Cases (*n* = 95,307)	Controls (*n* = 95,307)	*P*-value
Current age (mean)	42.2 (14.9)	52.8 (20.2)	<.0001	42.2 (14.9)	42.2 (14.9)	
Demographics, *n* (%)						
White	43,309 (45.4)	5,074,673 (65.6)	<.0001	43,309 (45.4)	43,348 (45.5)	.86
Black	32,039 (33.6)	986,109 (12.7)	<.0001	32,037 (33.6)	32,108 (33.7)	.73
Unknown race	13,321 (14.0)	1,020,372 (13.2)	<.0001	13,321 (14.0)	13,269 (13.9)	.73
Female	71,074 (74.6)	4,177,386 (54.0)	<.0001	71,072 (74.6)	71,091 (74.6)	.92
Male	21,326 (22.4)	3,137,300 (40.5)	<.0001	21,326 (22.4)	21,329 (22.4)	.99
Diagnoses, *n* (%)						
Primary hypertension	25,656 (26.9)	2,453,520 (31.7)	<.0001	25,655 (26.9)	25,667 (26.9)	.95
Hyperlipidemia	20,513 (21.5)	2,569,945 (33.2)	<.0001	20,513 (21.5)	20,564 (21.6)	.78
Diabetes mellitus	15,633 (16.4)	923,040 (11.9)	<.0001	15,631 (16.4)	15,520 (16.3)	.49
Nicotine dependence	20,816 (21.8)	626,681 (8.1)	<.0001	20,814 (21.8)	20,896 (21.9)	.65
BMI ≥ 30	46,229 (48.5)	2,194,553 (28.4)	<.0001	46,227 (48.5)	46,354 (48.6)	.56

*HS*, Hidradenitis suppurativa.

**Table II:** Association of OSA with HS

TriNetX	Unadjusted risk ratio (95% CI)	*P*-value	Adjusted risk ratio∗ (95% CI)	*P*-value	Absolute risk†, HS, *n* (%)	Absolute risk†, control, *n* (%)
Obstructive sleep apnea	2.02 (1.96-2.08)	**<.0001**	1.46 (1.42-1.50)	**<.0001**	12,889 (13.5)	8832 (9.3)

*P*-value of <.0001 demonstrates a statistically significant assocation between OSA and HS.

*HS*, Hidradenitis suppurativa; *OSA*, obstructive sleep apnea.

∗ Adjusted by matching for other risk factors for developing sleep apnea: obesity, smoking, hypertension, hyperlipidemia, and diabetes.

† Values listed are from the adjusted risk cohorts.

Our findings align with limited prior evidence. One case-control study (*n* = 23,767) from the National Inpatient Sample demonstrated significant HS-OSA association (OR = 1.274) after adjusting for age, sex, smoking, and obesity,^3^ which we extend by also controlling for hypertension, hyperlipidemia, and diabetes. Another matched case-control study (*n* = 1653) with All of Us reported a threefold increased OSA risk in patients with HS.^2^ Additionally, Wertenteil et al found elevated OSA incidence among patients with HS (3.5% vs 2.5% in controls; aOR 1.45), supporting potential temporality.^5^ Our large EHR-based study includes 1 of the largest sample sizes to date investigating the HS-OSA association, demonstrating significantly greater OSA prevalence in HS, even after adjusting for obesity and other potential co-linear diseases. Shared systemic inflammation and tissue laxity may contribute to pathophysiology, though further prospective research is needed. Limitations include residual confounding and incomplete EHR data. Some variables (eg, smoking, pain) were inconsistently captured, and other potential confounders (eg, opioid/alcohol use, Down syndrome) were excluded. Finally, our retrospective design limits causal inference. Nonetheless, reevaluation of HS screening recommendations for OSA may be warranted, especially among nonobese patients with HS. Given the morbidity of untreated OSA, future guidelines may consider incorporating simple evidence-based inquiries of sleep quality in patients with HS and consider referral for polysomnography where indicated.

## Conflicts of interest

Alexandra Charrow is a PI for clinical trials with Insamed, Incyte, and Sonoma. She serves on the BOD of the HS foundation. Her career is funded through a CDA through the Dermatology Foundation. She has served on advisory boards for Avalo, UCB, Incyte, Wedbush, and previously Novartis. Patil, Rohan, Sakunchotpanit, Venkatesh, Milosavljevic, and Nambudiri have no conflicts of interest to declare.
